# Assessment of carbon in woody plants and soil across a vineyard-woodland landscape

**DOI:** 10.1186/1750-0680-6-11

**Published:** 2011-11-09

**Authors:** John N Williams, Allan D Hollander, A Toby O'Geen, L Ann Thrupp, Robert Hanifin, Kerri Steenwerth, Glenn McGourty, Louise E Jackson

**Affiliations:** 1 University of California, One Shields Ave., Davis, CA 95616, USA; 2 Fetzer Vineyards, 13601 Old River Road Hopland, CA 95449, USA; 3 USDA Agricultural Research Service, 595 Hilgard Lane, Davis, CA 95616, USA; 4 University of California Cooperative Extension, 890 North Bush Street, Ukiah, CA 95482-3919, USA

**Keywords:** aboveground carbon, agriculture, allometric equation, biodiversity, ecosystem services, GIS, habitat, organic farming, sequestration, soil carbon

## Abstract

**Background:**

Quantification of ecosystem services, such as carbon (C) storage, can demonstrate the benefits of managing for both production and habitat conservation in agricultural landscapes. In this study, we evaluated C stocks and woody plant diversity across vineyard blocks and adjoining woodland ecosystems (wildlands) for an organic vineyard in northern California. Carbon was measured in soil from 44 one m deep pits, and in aboveground woody biomass from 93 vegetation plots. These data were combined with physical landscape variables to model C stocks using a geographic information system and multivariate linear regression.

**Results:**

Field data showed wildlands to be heterogeneous in both C stocks and woody tree diversity, reflecting the mosaic of several different vegetation types, and storing on average 36.8 Mg C/ha in aboveground woody biomass and 89.3 Mg C/ha in soil. Not surprisingly, vineyard blocks showed less variation in above- and belowground C, with an average of 3.0 and 84.1 Mg C/ha, respectively.

**Conclusions:**

This research demonstrates that vineyards managed with practices that conserve some fraction of adjoining wildlands yield benefits for increasing overall C stocks and species and habitat diversity in integrated agricultural landscapes. For such complex landscapes, high resolution spatial modeling is challenging and requires accurate characterization of the landscape by vegetation type, physical structure, sufficient sampling, and allometric equations that relate tree species to each landscape. Geographic information systems and remote sensing techniques are useful for integrating the above variables into an analysis platform to estimate C stocks in these working landscapes, thereby helping land managers qualify for greenhouse gas mitigation credits. Carbon policy in California, however, shows a lack of focus on C stocks compared to emissions, and on agriculture compared to other sectors. Correcting these policy shortcomings could create incentives for ecosystem service provision, including C storage, as well as encourage better farm stewardship and habitat conservation.

## Background

Worldwide, landscape mosaics that include forests and perennial agricultural production systems have benefits in terms of stored C and biodiversity protection [1-3]. Integration of forest and agricultural ecosystems into complex landscapes also increases the provision of other ecosystem services, including pest management, nutrient retention, erosion control, and water quality [4-7]. In regions where intensive agriculture becomes economically successful, loss of natural ecosystems is often rapid, and people forego the ecosystem services that are provided when these systems are included in the landscape mosaic [8,9]. In such situations, the existing incentives may not be sufficient to make complex landscape mosaics economically viable. For sustained productivity and environmental health, incentive mechanisms will need to be augmented or developed to support the joint management of natural and agricultural ecosystems in the same matrix. To that end, methods are needed to evaluate C stocks and biodiversity of woody species as baseline properties of agricultural landscapes that are composed of many types of ecosystems.

Forests are storehouses of both biodiversity and C [10-13] compared to most agricultural systems, which are typically rebuked for their role as emitters of greenhouse gases (GHG) from soil and drivers of forest loss [14-17]. Relatively little research has been conducted, however, to assess the potentially beneficial role that agricultural landscapes can play when managed as complex systems that harbor species and habitat diversity, preserve forested lands and sequester C, while supplying the essential provisioning services on which humanity depends [18-23].

Given the extent to which agriculture has transformed global terrestrial and aquatic ecosystems [24], and the likelihood that much of the land in agricultural production today will stay that way for the foreseeable future, there is an urgent need to promote multi-functionality in agricultural landscapes [25,26]. Especially in areas of high biodiversity, agricultural landscapes that contain a mosaic of ecosystems provide multiple ecosystem services with benefits that cross the boundaries of economic sectors and scientific disciplines, such as pollination, regulation of water quality and supply, and mitigation of GHGs [7].

In the species-rich Mediterranean-type ecosystems of the world, vineyard expansion poses a major threat to biodiversity and ecosystem integrity [27,28], potentially contributing to the loss of natural habitat, species diversity and to a net release of C into the atmosphere [29,30]. It is possible, however, for vineyard managers to balance crop production with habitat conservation and other ecosystem services [31,32]. In the winegrowing region of California--also widely recognized for species richness and habitat diversity [33,34]--some private landowners are making a concerted effort to maintain and/or restore natural plant communities as part of the vineyard landscape matrix. This study highlights one such example, examining the effect of stewardship efforts at a vineyard in Mendocino County, California, to maintain habitat diversity, safeguard C stocks, use organic practices, and minimize the C footprint across the winegrower's landholdings.

**Figure 1:**
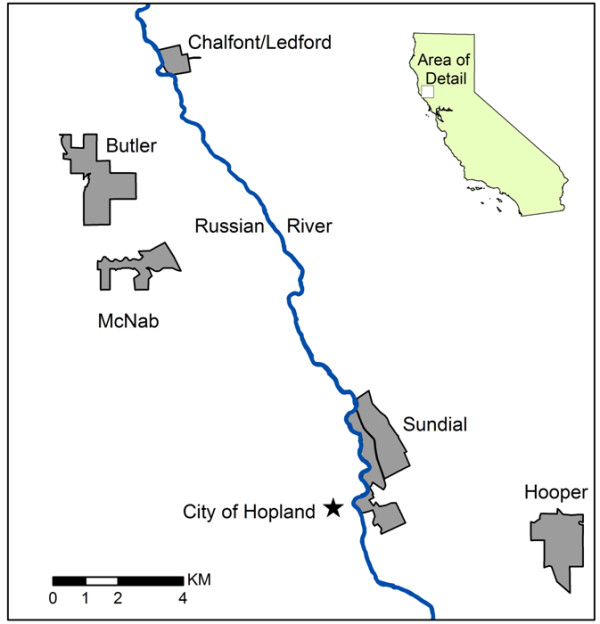
**Study site in Mendocino County, California (state shown in inset), with the location of the five wine grape-growing ranches (labeled) where carbon stocks were assessed for vineyards and adjoining wildlands**.

This study in northern California (Figure 1) represents a participatory approach between a commercial winegrower, Fetzer/Bonterra Vineyards, and university researchers with the goal of quantifying C stocks across a landscape mosaic of vineyards and wildlands on the owner's land. (Wildlands are defined here as habitat dominated by native vegetation that does not show obvious signs of degradation due to previous use, such as cropping, grazing or timber harvesting.) The specific objectives of the study were to: 1) estimate C stocks across the vineyards and various types of wildlands using field sampling and statistical models; 2) examine partitioning of aboveground and soil C stocks in vineyards and wildlands on ranches in different landscape positions; and 3) develop a methodology for estimating C stocks that is accurate, accessible in terms of complexity, and meets requirements for government-regulated C accounting programs. The central hypothesis is that the heterogeneous mixed vineyard-wildland mosaic considered in this study reflects variability in vegetation and C storage potential, and provides greater C stocks and biodiversity protection than vineyards alone. The study uses a geographic information system (GIS) to analyze how a suite of variables affects C stocks, including tree and shrub species data, topographic variables (slope, elevation, and aspect), maps of soil and vegetation types, and remotely-sensed spectral data. We refer to vegetation types as broad classes of wildland vegetation that were identified in the GIS. The study concludes with a discussion of C policy in California, and recommends that policy makers reconsider the importance of C stocks in agricultural systems for GHG mitigation as well as for improving farm stewardship, conserving wildlands, and/or incorporate planting of native trees and vegetation on farm properties.

## Results

**Figure 2:**
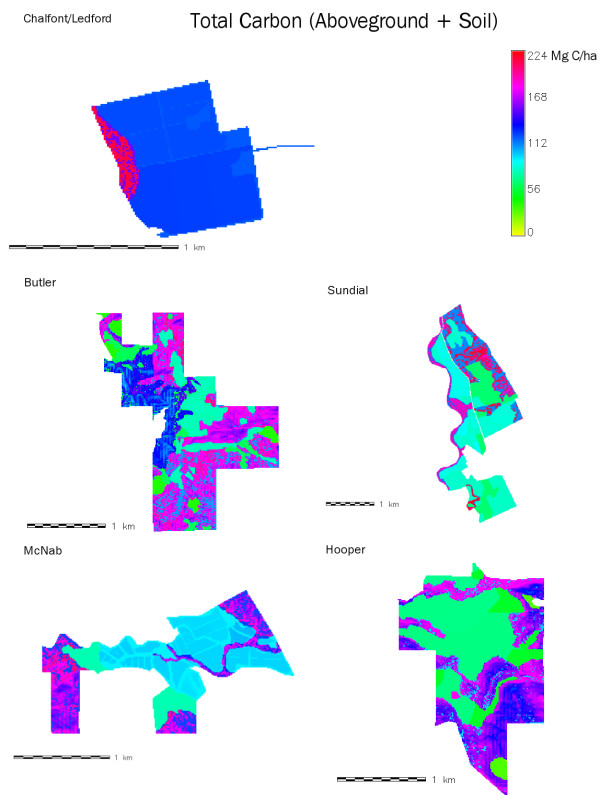
**Spatial representation of total carbon stocks in aboveground wood and soil (to 1 m depth) for the five ranches considered in this study**. Counterclockwise from top left, they are: Chalfont/Ledford; Butler; McNab; Sundial; and Hooper.

Aboveground woody C stocks were greater in wildlands than in vineyards (Table 1). On average, forested wildlands had 45% more total C/hectare (ha) than vineyards. That number breaks down to approximately 12 times more aboveground woody C and 6% more soil C in wildlands than in vineyards. Soil organic carbon (SOC) varied substantially within land-use types (i.e. vineyards or wildlands) across ranches, though not as much as aboveground C. Variation in total C/ha within ranches was greatest for those ranches with more varied topography and vegetation, such as Butler and Hooper (Figure 2). For a given ranch and a given C source (soil or aboveground), wildlands were consistently higher in C stocks than vineyard blocks, but variation within vegetation types was greatest in upland forests and least within riparian corridors.

**Table 1:** Carbon by reservoir for each of five ranches assessed by the two land cover types

Ranch	Vineyards			Wildlands		
	Soil Mg C/ha	AG-Wood Mg C/ha	Total Mg C/ha	Soil Mg C/ha	AG-Wood Mg C/ha	Total Mg C/ha
Chalfont/Ledford	118.7	3.6	**122.3**	132.7	14.0	**146.6**
Butler	76.0	2.3	**78.3**	87.6	47.6	**135.2**
McNab	92.3	4.5	**96.8**	106.8	19.2	**125.9**
Sundial	80.0	4.1	**84.1**	91.8	22.8	**114.7**
Hooper	68.0	0.7	**68.7**	83.8	34.3	**118.1**

Average	84.1	3.0	**87.1**	89.3	36.8	**126.1**

Std Dev	6.6	0.48	**7.0**	14.5	8.6	**22.9**

Estimates were derived by fitting multivariate linear regressions to C values for sample plots (aboveground) and measured soil C (from soil pits) using environmental variables such as elevation, slope, solar radiation, soil taxonomy, normalized differential vegetation index (NDVI) and habitat type in a GIS platform (see methods). Soil C is to 1 m depth; AG = aboveground; aboveground C on wildlands excludes grasslands.

**Figure 3:**
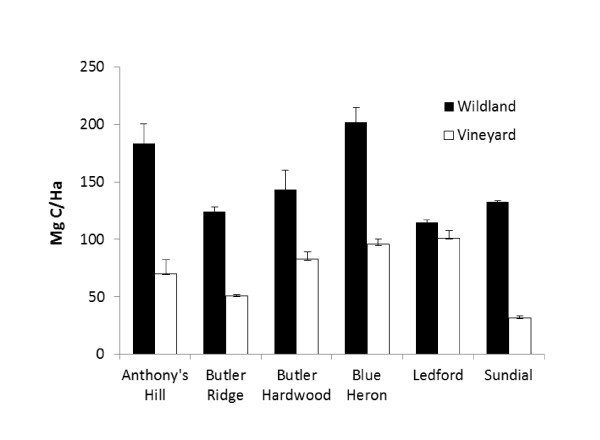
**Comparison of wildlands (dark bars) and vineyards (hollow bars) for carbon in the top meter of soil for paired (adjacent) soil pits at different locations across the study site (horizontal axis)**. Carbon values are extrapolated to per ha estimates. Error bars represent standard error based on four measurements per site.

Although SOC in the organic vineyard blocks was relatively high by temperate conventional cropping standards [35,36], SOC was nevertheless consistently higher in wildlands than in vineyards for a set of paired soil pits (i.e., adjacent pits on the two land-use types) and for ranch-wide estimates based on the averages of all wildland and vineyard pits (Figure 3; Table 1). Overall, SOC varied 1.7-fold among ranches, with the Chalfont/Ledford ranch characterized by alluvial soils from the Russian River having the highest C content, and the Hooper ranch characterized by loamy upland soils with the lowest C content. Within vineyard tracts, there were no correlations found between SOC and vine age, slope, aspect or elevation. There were no detectable effects of management practices (such as tillage schedule, use of compost, or cover crop mix) on C in the top 15 cm of soil (the soil layer where management practices are most likely to have an effect), as revealed by a survey instrument that consisted of summarizing each block's management practices over the past five years with a simple scoring system (Additional File 1: Appendix 1). The maximum possible score was 6, and the mean score was 2.2, with the majority of blocks having two or more management practices applied to increase SOC and/or boost nutrient retention.

**Figure 4:**
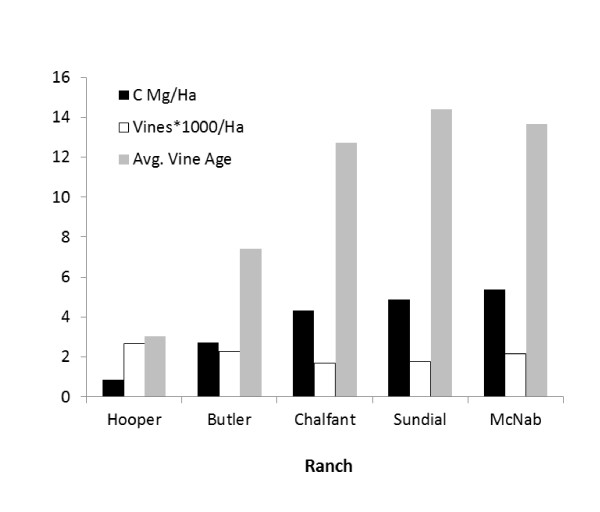
**Aboveground carbon in grape vines on each ranch as a function of vine age (in years) and number of vines per ha**.

Per ha, the aboveground woody C in vineyards varied more than 6-fold among ranches, and was a direct function of vine age and number of vines per ha (Figure 4). Aboveground woody C in wildlands varied 3.4-fold among ranches (Table 1). More informative, however, was the variation in aboveground C in wildland plots within ranches, where C differences between sample plots varied as little as 2-fold and as much as 120-fold (Additional File: Appendix 2). At the Chalfont/Ledford ranch, where the within-ranch wildland C varied the least, only valley riparian vegetation was present. Here, woody plant diversity was lowest with a total of only five tree species (dominated by a maple (*Acer*) - cottonwood (*Populus*) association), and the vegetation structure was defined primarily by a an understory layer of immature trees 3-8 m tall, and an overstory layer 17-22 m tall. By contrast, at the Butler ranch where between plot variation was greatest, at least 18 woody tree and shrub species were present. All of the vegetation categories were represented, and habitats ranged from manzanita (*Arctostaphylos*) - dominated chaparral 2-3 m in height, to oak (*Quercus*) woodland, to closed-canopy mixed conifer-hardwood forest 15-25 m in height. The Hooper, McNab and Sundial ranches were intermediate between these extremes, being composed mostly of closed-canopy mixed hardwood stands 13-18 m in height, and interspersed with patches of grassland, oak woodland and valley riparian vegetation (Additional File 1: Appendix 2).

Modeling of aboveground woody C in the entire wildland acreage was done using multivariate linear regression with up to four environmental variables (topography, habitat, soil and ranch). The optimal model based on the lowest Akaike Information Criterion (AIC) value [37] included only ranch and habitat type as predictor variables (R^2 ^= 0.131; p = 0.303). Thus, a separate equation was used to estimate aboveground C across the landscape at each ranch. An evaluation of the predictive ability of the same model using training (70%) and testing (30%) data sets yielded a weak relationship (R^2 ^= 0.16) across the entire land area of the five ranches.

Multivariate linear regressions relating estimated C and basal area of woody biomass to environmental variables for individual species generally yielded better correlations than the all-species model. Table 2 shows the fit for the 14 most important species in terms of overall contribution to biomass on the five ranches, which accounts for 95% of the biomass sampled. Eight of these species-specific regressions show a level of significance of p < 0.05; these eight species account for 54% of the biomass measured in the sample plots.

**Table 2:** Coefficients of determination (R^2^) for species-specific regression equations fitting aboveground woody carbon and basal area to topographic, vegetation, soil and landscape variables (see methods)

Biomass Ranking	Species	**Carbon - R**^**2**^	**Basal Area - R**^**2**^
1	*Quercus kelloggii *	0.203	0.262*
2	*Q. wislizenii*	0.103	0.118
3	*Q. lobata*	0.143	0.176
4	*Q. douglasii*	0.385***	0.379***
5	*Arbutus menziesii *	0.279**	0.300**
6	*Acer negundo*	0.307**	0.359***
7	*Populus species *	0.307**	0.350***
8	*Salix species*	0.515***	0.538***
9	*Umbellularia californica*	0.113	0.119
10	*Q. species*	0.103	0.069
11	*Q. chrysolepis*	0.217	0.221*
12	*Pseudotsuga menziesii*	0.051	0.079
13	*Aesculus californica *	0.186	0.186
14	*Alnus rhombifolia*	0.278**	0.310**

p-values: * < 0.05; ** < 0.01; *** < 0.001

Listed by rank are the 14 tree species that contribute most to total aboveground woody biomass estimated for sample plots in wildlands surrounding vineyards on five ranches in Mendocino County, California.

On average, each of the ranches had 4.6 of the seven habitat types that had been identified by the PAM analysis at the onset of the project (Table 3). Across the five ranches, 28 woody species were sampled, representing 17 genera and 12 families (Additional File 1: Appendix 3). In terms of taxonomic richness, the family Fagaceae and the genus *Quercus *within it were the most important, with 9 and 8 species, respectively. The average number of woody wildland species was 14.4 per ranch (std. dev. = 5.2), and 3.1 per 10 × 30 m plot (std. dev. = 1.2). While not quantitatively assessed, an informal visual assessment of herbaceous plants revealed that all ranches except Chalfont/Ledford had substantial diversity in annual and perennial herbs as well as healthy communities of native perennial grasses mixed in with communities of European annual ruderal grasses. The diversity of soil types was high, as a total of eight great groups were sampled. These were grouped according to topographic features as follows: uplands (Haploxeralfs, Argixerolls, Palexeralfs, and Xerorthents); valleys (Haploxerolls, Xerofluvents, and Haplaquolls); and terraces (Palexeralfs).

**Table 3:** Land cover types found on the five wine grape-growing ranches in Mendocino County, California, considered in this study

Number of Vegetation Plots	Number of Soil Pits	Habitat Type	Elevation (meters)	Slope (degrees)	Solar Radiation (W-hr/m^2^/yr)
0	19	Vineyard I	173	0.7	2.18*10^6^
0	6	Vineyard II	572	10.7	1.99*10^6^
0	2	Annual Grassland	213	5.0	2.27*10^6^
17	1	Valley Riparian	152	0.4	2.18*10^6^
39	2	Mixed Hardwoods I	534	21.3	1.97*10^6^
26	13	Mixed Hardwoods II	225	10.7	2.16*10^6^
11	1	Mixed Conifer-Hardwood	407	25.8	2.06*10^6^

The classification process consisted of a partitioning around mediod (PAM) analysis, which used 1013 random points and binned them into similar groups based on four variables: land cover; elevation; slope; and solar radiation (see methods).

Analysis of C and species diversity in multidimensional space using NMDS ordination revealed slope and elevation to be the two strongest environmental variable vectors affecting C (Additional File 1: Appendix 4). Soil organic C was a strong component that loaded in the opposite direction of elevation, consistent with the fact that organic matter is lost during erosion of upland soils and accumulates in bottomlands. The ordination, which had only moderate stress (12.3%), also showed species and plots oriented along a primary axis characterized by proximity to water, with riparian species such as alders (*Alnus sp*.) and willows (*Salix sp*.) on ranches like Sundial (near the Russian River) on one end, and drought tolerant species such as manzanitas (*Arctostaphylos spp*.) and toyon (*Heteromeles arbutifolia*) on ranches like Hooper (upland hills and small valleys) at the other end. No other variables appeared to impose obvious structure in the ordination.

## Discussion

### Research Implications

The most salient results of this research for C stocks are that: 1) per ha, substantially more C was stored in the top m of soil (not including roots) than in aboveground biomass; and 2) as expected, both the above- and belowground components of wildlands contained consistently more C per ha than vineyards. Within-ranch SOC comparisons showed wildlands averaged 16% more C per ha than vineyards--a value supported by the paired soil pit comparisons (Table 1; Figure 3). Among wildland vegetation types, valley riparian vegetation was associated with the highest C stocks, the major component of which came from soil. This trend may be explained by long-term deposition of organic material along the floodplains of the Russian River and its tributaries [38]. For non-riparian vegetation where SOC was more variable, closed-canopy mixed hardwood forest (e.g., the Butler and Hooper ranches) made the greatest contribution to C stocks. For vineyard tracts, the differences in aboveground C were explained by the age of the vines, which was tightly correlated with biomass and C content (Additional File: Appendix 5). Even the largest vines, however, had only about one-fourth of the woody biomass per ha of the adjacent wooded wildlands.

The aim of this study was to determine whether vineyard landscapes managed as mosaics of vines and wildlands could yield ecosystem benefits, specifically higher C stocks, superior to that of vineyard blocks alone. Considered this way, the results strongly support our starting hypothesis and suggest that including closed canopy forest and forested riparian corridors as part of the wildlands component may be particularly good ways to increase overall C stocks. Similarly, the planting of native trees and habitat (in corridors or hedgerows) in farming systems can have benefits for winegrowers and other agricultural operations. Additional research examining C dynamics within soil units and vegetation types would be helpful for quantifying rates of C accumulation, as well as for identifying the causal mechanisms for the C differences that were observed [e.g., [36]].

If maximizing C stocks is a management objective, then the conversion of intact forest lands should be minimized, as these lands consistently store more C per ha than vineyards (or than agricultural systems in general [39]). Likewise, practices that conserve soil organic matter and reduce soil disturbance will protect the largest single reservoir of C in the farm system [40,41]. We recognize, however, that these are rarely the explicit objectives of land managers. Instead, as managers map out or reconfigure the array of vineyard rows on their lands, they should look for opportunities to conserve and/or restore existing wildlands and minimize soil disturbance. Maintaining wildland vegetation on steep slopes or along stream corridors, for example, can reduce erosion in California's climate--thereby protecting soil resources and safeguarding water quality--as well as boost C stocks [42,43]. Evidence also suggests that in places where vines are not appropriate but where wildlands do not exist, forest restoration and hedgerow plantings may offer additional ways to increase C stocks in the landscape [[43]; see also http://privatelands.org/FSP/NRCS_conservation_practices.htm, [44]].

Because the greatest quantities of C stocks in the study landscape were below ground, soil management is of major concern. The survey instrument to determine whether different organic management practices yielded similar scores because most tracts had multiple management interventions to achieve a variety of outcomes, including nutrient retention, weed suppression and erosion control. Organic management practices have been shown to increase soil C [45-47], and reducing soil disturbance has been shown elsewhere to decrease C loss [15,48]. The small differences in soil C between paired woodland and vineyard sites, compared to another study that compared woodlands to conventional vineyards [29], suggest that the no-till, cover cropping practices and organic matter management used in this study system are conducive to soil C retention. Future, small-scale manipulations with control plots could be used to determine the effect of a specific interventions to improve soil C, but would likely require several years to show differences [41,49,50].

### Modeling Challenges and Potential Solutions

The methods used and the subsequent results from this research support a mosaic model for vineyard management, where management objectives and landscape variability determine the amount and configuration of vine tracts and wildlands. It was the landscape variability--whether in terms of slope, aspect, soil quality or species composition--that also presented the greatest challenges to modeling C in this study. Our use of vegetation-based habitat types to classify C stocks in the landscape was a good starting point for modeling aboveground C stocks. Woody species importance and composition frequently differed substantially within the same habitat type, however, especially for the mixed hardwood categories (these associations are known to contain numerous vegetation series, see [34]). Thus, while single-species models produced robust predictive regressions using environmental variables, this approach often did not improve prediction of C in the landscape because of the numerous species involved. The high variability of woody plant diversity at sampled sites both within and among ranches made it challenging to correlate C with environmental variables such as slope, aspect, and soil characteristics. To not consider the variability in plant diversity, however, would have been to ignore the complex patterns that shape the landscape and the C contained therein. The models developed in this study therefore demonstrate the importance of a comprehensive approach that combines field data and remotely-sensed environmental variables for predicting C in the landscape. Future efforts can build on these models to test the utility of including a second tier of variables, such as dispersal patterns, evolutionary history and phylogeny, biogeography and human influences (see [51]).

The variability in species composition and distribution within habitat types, as well as the diversity of soil types across even small areas, highlight the need to refine models to address heterogeneity for assessing C stocks. Some assessments that have assigned a generalized C value for a given habitat type (e.g., riparian forest vs. oak woodland) or even an entire forest seem to offer a pragmatic solution for C estimation (e.g. Brown *et al*. [52]). The present study suggests, however, that such approaches do not give acceptable resolution for the complex Mediterranean-type ecosystems evaluated here. Heterogeneity between stands means that extrapolating a generalized C value for a habitat type to a regional scale will result in an amplification of error, especially when GIS data are used without field verification [53]. In addition, specific projects must consider heterogeneity in C stocks for addressing appropriate mitigation of the GHG emissions that occur when wildlands are deforested for agricultural production [54].

The C assessment methods used in this study were based on recommendations outlined in the Climate Action Registry (CAR) protocol for inventorying C in California forests [55,56]. While the protocol has been updated twice since our assessment began [57,58], additional improvements are both necessary and ongoing. Our results highlight two areas that can be targeted for improving future woody plant C estimates: 1) extrapolating aboveground woody biomass from field measurements using allometric equations based on metrics such as diameter at breast height (DBH, where height = 1.3 m) and tree height; and 2) understanding which environmental variables best explain the variation in aboveground woody biomass and obtaining values for these variables at the appropriate spatial scale.

Accurate modeling of aboveground woody C in a mosaic of mixed-age, mixed-species vegetation remains a challenge. A broad suite of allometric equations were used in this study to differentiate between the woody species found in the landscape and to account for the effect of those differences on biomass. While this method represents a best-available-information approach to estimating C, a more complete set of allometric equations relating tree diameter to volume and biomass is needed. Many of the equations available were either not specific to the study species or were based on inappropriate subsamples of study species (e.g., subspecies from a different part of a broad geographic range or limited to size classes that may not extrapolate well to the size classes encountered here). As a result, several equations were generalized to genus, family, growth form or foliage type (e.g., evergreen or deciduous) [59]. Equations do not currently reflect the natural variation in site conditions and growth form among individuals. Measured differences in architecture among species, subspecies, regional races, or cultivars vary from 5 to 300% [60-63]. While more sample plots would arguably improve estimation power, such efforts increase the time and cost of research. The challenge is to find ways to use species-specific equations to incorporate more detail on vegetation variability without dramatically increasing the number of sample plots. The use of remote sensing for data collection may help in this regard. While satellite imagery is generally too coarse for the types of analyses conducted here, techniques such as LiDAR may improve wood volume assessments [64,65]. Currently, costs to obtain such data are high, however, and such techniques will still require ground truthing of species diversity and species-specific wood density to generate accurate data on C stocks.

Greater spatial resolution of soil taxonomic classifications and related maps across the landscape would improve accuracy in estimating soil C. Given the importance of soil C in our findings, it is unfortunate that the scale of soil mapping and intensity of investigation in forests and rangeland are typically less than agricultural areas [66]. We found that map units often contained more than one component (soil type) but lacked explicit delineation between components, resulting in a loss of important information about soil variation. Also, SOC measurement down to 1 m depth is labor intensive and costly, so the methods used here are not practical for most landowners. Updated mapping of wildland areas by the National Cooperative Soil Survey would better represent the edaphic complexities of the landscape, improve the accuracy of SOC estimation and reduce costs for a broad user base.

For both the above- and below-ground components of C, there are also publicly available resources that assist in C accounting and on-farm management. COMET-VR from the United States Department of Agriculture http://www.comet2.colostate.edu/about/ stands out as a useful tool for on-farm evaluation and management related to C sequestration. Likewise the Global Research Alliance http://globalresearchalliance.org/ offers a forum for sharing of research, data and expertise to reduce C outputs and improve efficiency in agricultural systems. Undoubtedly, numerous additional online resources also exist.

### Implications for Policy

The above suggestions for improving C stock estimation are based on the premise that regulating bodies will either make C accounting mandatory or provide incentives for maximizing C storage. At present, however, it is not clear that this is the case. While C accounting is a two-part issue made up of inputs (sequestration) and outputs (emissions of CO_2 _and other GHGs), currently, most regulation is focused on emissions. The California Air Resources Board, for example, is responsible for enforcing Assembly Bill 32, the state law that requires statewide reductions of GHG emissions to 1990 levels by 2020 [31]. The US Government has likewise taken an emissions control approach, as was highlighted in December 2009, when the Environmental Protection Agency declared CO_2 _and five other GHGs to be air pollutants subject to regulation. Although C offset markets in the US are currently voluntary, this action may pave the way for future compliance-based C markets. In California, viable voluntary C offset projects are those that qualify for one of three categories: reforestation; improved forest management; or avoided conversion. The first two seek to increase forest cover by planting or management techniques, while the third is for forested land that is at risk of conversion and meets the requirement of additionality.

Additionality refers to the stipulation that for a parcel of forest to qualify for credit, it must be at imminent risk of conversion to non-forest [58]. The owner must also prove that s/he is not keeping the parcel forested because of an existing easement or set-aside (as an example, we note that part of the study area for the research presented here is excluded from C credits because it is in a conservation easement). While additionality is a common element of regulatory frameworks because it prevents landowners from getting credit for land that is either not at risk of conversion or would yield no net reduction in emissions [12], it may lead to perverse incentives such as preventing owners from entering into conservation easements or encouraging them to clear forested land just so it can be replanted and qualify for reforestation.

Because the current regulatory focus in the US is on pollution rather than storage, most of the funds go toward curbing emissions. In California in 2006, transportation, energy production and industry accounted for more than 80% of annual GHG emissions, whereas agriculture collectively contributed only 6% (of which livestock made up more than 50% [67]). Furthermore, the average farm size in California in 2007 was 313 acres (127 ha) with only 5.5% of farms larger than 1000 acres (405 ha) [68]. Given there are more than 25 million acres (10 million ha) of farmland in the state, this makes transaction costs very expensive.

Implementing an incentives program is only worthwhile if it results in pronounced increases in C stocks (relative to emissions) or in other economic benefits. With respect to the latter, one possibility is quantifying C stocks together with other ecosystem services, such as water quality and storage capacity, soil erosion and nutrient run-off control, and threatened or endangered species habitat protection (e.g., stream habitat for salmon). This approach is increasingly discussed in the environmental economics literature, though examples of its implementation remain few [69,70]. Nevertheless, farmers who participated in programs that provided rewards for responsible management of multiple ecosystem services might find the transaction costs worthwhile in terms of both the on- and off-farm goods received.

## Conclusions

The company (Fetzer/Bonterra Vineyards) that purchased the ranches considered in this study did so with the intention of growing organic grapes. The subsequent decision to maintain a large fraction of that land in natural habitat was based not on an economic rationale, but rather on an environmental ethic to combine wine production with conservation of the landscape's natural integrity. This approach also included a series of sustainability measures (e.g., 3^rd ^party certification, solar power generation, reduced packaging, GHG emission reductions through fleet fuel efficiency, etc.). The company did not attempt to quantify either the ecosystem services generated by this type of land management or the biological diversity protected as a result. The present research suggests, however, that this type of multifunctional land management generates outputs, such as increased C stocks and biodiversity protection, that are not necessarily included on a company's ledger sheet. Additional ecosystem services were not measured as part of this study, but were likely generated from this management approach, such as prevention of soil loss and water quality protection, and would be worth quantifying for associated benefits. If payments for ecosystem services become a reality, a complete accounting of such benefits may provide further justification for a multifunctional management approach.

Although agriculture represents a small percentage of overall GHG emissions, the agricultural landscape that includes perennial woody crops such as vineyards, soil, and wildland vegetation in the matrix around crops represents a major source of potential C storage (or release). Based on the example of a mixed vineyard/woodland landscape, our research suggests that the C storage potential and habitat conservation value of wildlands within the agricultural matrix may be much greater than that of cultivated land alone. As other ecosystem services provided by mixed agricultural systems becomes better quantified [7,26,71], the natural habitat and biodiversity of these systems may be increasingly valued by farmers and society alike. With a more thorough accounting system for the ecosystem services (including C storage) provided by agricultural landscapes, a regulation or an incentive program that encourages management approaches that promote C storage may become viable. Vineyard landscapes are often composed of a mix of agricultural and non-agricultural land that varies in the above- and belowground C stocks it contains. To maximize their C stocks, landowners need a way to assess their actual and potential C stocks, and accurate ways to measure them. The complexity of the Mediterranean-type biome provides a good context for addressing these issues.

## Methods

This study estimates C stocks on the five ranches (Butler, Chalfont/Ledford, Hooper, McNab, and Sundial) that produce organic grapes for the Bonterra label of Fetzer Vineyards, located in Mendocino County, California, USA (39.0° latitude, -123.1° longitude). The ranches, which are scattered across the Russian River Valley (Figure 1), vary in topography, size, and the number and extent of different habitat types. In total, they comprise 1149 ha: 350 ha in vineyards; 400 ha in forested wildlands (woodlands or forests with at least one tree or woody shrub per 10 × 10 m), and 449 ha in grasslands (wildlands without trees). The ranches were converted to organic management during the late 1980s, gaining organic certification by California Certified Organic Farmers between 1991 and 2005. The salient management practices in these organic vineyards include planting of cover crop mixes, use of compost, water and soil conservation measures, elimination of synthetic pesticides and fertilizers, minimum tillage between vine rows (~15 cm depth), mechanical weed removal under vines, conservation of biodiversity, and planting of hedgerows or habitat corridors in and around the vineyards. Although the inclusion of wildlands as part of each ranch occurred as a condition of the sale (i.e., it did not represent an explicit management objective on behalf of the buyer), the current owner has actively protected the majority of these lands for conservation and ecosystem service values.

### Selection of sites

To select vegetation and soil sampling locations, we stratified the landscape according to habitat type to capture the variation in topography, land use, and vegetation on the five ranches. Data layers were input into a geographic information system (GIS) for four variables: land cover (according to the California Wildlife Habitat Relationships system, http://www.dfg.ca.gov/biogeodata/cwhr/); elevation; slope; and clear-sky solar radiation summed over a calendar year and modeled from a digital elevation model [72]. A random point generator populated the classifiable area with 1013 points, for which a matrix was created with the four GIS variable values. Gower's dissimilarity algorithm [73] generated dissimilarity distances between all points based on the numeric and categorical data. With this pre-selected number of random points on the landscape, an unsupervised learning technique grouped the points into seven classification units according to shared features drawn from the layers [74]. A cluster analysis, called Partitioning Around Medoids (PAM), used the distance matrix to group the points into the following habitat classification units: vineyard I on flat lowlands; vineyard II on uplands; valley riparian; mixed hardwoods on moderate-sloped, lower elevation lowlands; mixed hardwoods II on steeper-sloped, higher elevation uplands; mixed hardwood-conifer on steeper-sloped uplands; and annual grasslands. These clusters formed the basis for the stratified field sampling conducted on the five ranches.

Because the focus was on woody C (herbaceous vegetation does not qualify for consideration in C credit programs because the decomposition, or turnover rate, is considered too rapid to add any long-term sequestration value), and because vineyard C was measured separately (see below), no vegetation sampling was conducted on vineyard or grassland habitat types. For the four remaining habitat types, the PAM clusters were used as the basis for selection of the vegetation sampling sites (Table 3), with the goal of sampling a habitat type at a frequency consistent with its importance in the study area.

### Aboveground C stocks in vineyards

The aboveground C in the wood of grape vines was estimated for all tracts (management units) on the five ranches using the age and number of vines per tract (Additional File: Appendix 5). Vine wood volume was estimated based on vine age, which was calculated from a regression analysis based on samples of different ages. For each sample, measurements were taken for main trunk height, main trunk diameter at 0.5 m above the ground surface, cordon lengths from the main trunk, and a standard estimate of cordon diameter using age (2.5 cm of growth in the first five years, then 0.25 cm each additional year; D. Koball, Fetzer Vineyards, personal communication). Vine trunks and cordons were assumed to be straight cylinders of constant diameter. From these measurements, we generated the following regression equation (1), where age is the number of years since grafting the scion:

(1) $$\text{Wood volume}=179.19*({\text{age}}^{1.3303})){\text{ R}}^{2}=0.84$$

This equation was derived by fitting a power function to the relationship between vine age and aboveground wood volume for 29 vineyard tracts (Additional File 1: Appendix 5B; Equation 1 has not been evaluated for vines older than 23 years, the maximum age of vines in this study). Vine biomass was then calculated by multiplying volume by wood density. Vine wood density was based on an analysis of Chardonnay vines on one of the ranches and given as 0.95 g dry weight/cm^3 ^fresh volume (J. Nosera and G. McGourty, unpublished data); no other values are available for wine grapes. Carbon content for vine wood (and wildland wood) was estimated as 50% of dry weight [75,76].

### Aboveground C stocks and species diversity in woody plants in wildlands

Ninety-three sample plots were located by first identifying the different habitat types for each ranch using the PAM cluster analysis (Table 3). The habitat polygons were then evaluated to note variation within type, and enough sample sites were identified to capture the major differences in vegetation, with a minimum of three samples located per habitat type per ranch, provided sufficient space. Each plot consisted of three 10 × 10 m subplots laid out in a line (i.e. 10 × 30 m). For each plot, the DBH of all live trees and shrubs over 5 cm DBH within the plot boundary were recorded along with the species identity. Because field data were collected during winter months when leaves and fruit were sometimes absent, some individuals could only be identified to the genus level (tree habit, attached dry leaves, bark, and bud scars were used to identify individuals when other key vegetative or phenological material were absent). Biomass volume of downed woody debris and standing deadwood (snags) was estimated for logs ≥ 10 cm in diameter and 1 m in length, and for snags ≥ 10 cm DBH. Volume was estimated from DBH measurements using published allometric equations for individual species found in sample plots [59,77,78]. When a species-specific equation was not available, the equation used was first that of the genus, then family, then the most morphologically similar species for which there was an equation (Additional File 1: Appendix 3). Published species-specific values for wood density (or generic values where species data were not available) were used to calculate woody biomass of the standing aboveground woody vegetation [59].

### Soil C in vineyards and wildlands

Soil samples were collected from 44 soil pits (19 in vineyards, 25 in wildlands), including six paired sites, where two adjacent pits were dug in a vineyard and in a wildland. The soil pit locations were determined using the PAM classification and by consulting maps from the USDA Soil Survey Geographic Database (SSURGO; http://soils.usda.gov/survey/geography/ssurgo/). A soil profile description was made at each site to ensure that each of the soil great groups mapped on a given ranch was represented.

Soil pits were dug with a backhoe or by hand to ≥ 1 m in depth, and the coordinates of each pit were input into a GIS. Soil samples were collected from four depths at each pit: 0-15 cm; 15-45 cm; 45-75 cm; and 75-100 cm. For each sample, bulk density was estimated by weighing clods coated in paraffin and then submerging them in a volumetric flask to measure their displacement [79,80]. Percentage of rock fragments in each sample was measured by sifting a known volume of soil through a 2 mm screen. The fraction of rock (> 2 mm) was subtracted from the soil volume. Dried, sieved, and weighed samples for each depth were analyzed for C content using an elemental combustion analyzer (Costech Analytical Technologies, Inc., Valencia, CA).

The soil pit descriptions were compared with those of the mapped soil units from SSURGO (summarized by the California Soil Resource Lab of the University of California, Davis, http://casoilresource.lawr.ucdavis.edu/drupal/). This comparison was done by importing the SSURGO soil unit maps into Google Earth http://earth.google.com and using the high-definition aerial photography to locate our soil pits within the mapped soil units. Descriptions of the soil map units were compared to respective soil pit descriptions to ensure that the generalized SSURGO descriptions were accurate. Because many SSURGO soil units were created from only a few sampled data points at regional scales (e.g., county or state-wide), we wanted to confirm the accuracy of this open-access data source. Soil from the pits was classified by the landform in which it was found (upland, bottomland, or terrace) and the soil great group to which it belonged using soil taxonomy techniques [81]. The resulting data were input as categorical environmental variables to inform regression analyses.

For the vineyard tracts, a management history for the past five years was obtained with a short survey instrument given to vineyard managers. The questions in the survey were about practices known to influence the rate of storage or loss of C in cultivated soil, i.e., frequency and extent of tillage, compost application and cover crops on each block (Additional File 1: Appendix 1). Responses to each question were coded as 1 or 0 (yes or no), and the scores for all questions summed up to give a cumulative management impact index where the greater the score, the greater the potential increase in soil C storage.

### Determination of C stocks across the landscape

To estimate total aboveground wood C across the wildland areas, a grid of 10 × 10 m cells was overlaid on the five ranches in a GIS. The woody biomass measured on the sample plots (Additional File 1: Appendix 2) was used in conjunction with a set of predictor variables from a variety of data sources, including satellite imagery, aerial photography, soil surveys, and digital elevation models to develop a set of multiple linear regression models. The variables were categorized into the following four groups: 1) topography - elevation, slope and solar radiation; 2) vegetation - summer normalized difference vegetation index (NDVI, based on Landsat 7 sensor platform); 3) soil - soil pH, soil organic matter, clay and sand (from SSURGO maps); and 4) landscape - ranch and landcover (vegetation) type (Additional File 1: Appendix 4). Fifteen variants of these four groups were used to model aboveground C, and the best fit model was selected based on the lowest AIC value [82]. The variable combination for the best fit model was then used to predict C values for unsampled grid cells by splitting the sample data into a training set (two-thirds) to build the model and a test set (one third) to evaluate how well the model predicts C for the withheld points.

In addition to the all-species model, multiple linear regression was used to model per species aboveground C across the landscape for the 14 species that contributed most to woody biomass in the sample plots (Table 2). Each model used the same four groups of variables as the all-species model, plus vegetation texture (a metric based on fine resolution, 2 m gray scale contrasts of aerial imagery from the National Agriculture Imagery Program of the USDA). This analysis was conducted to determine if any of the species taken independently demonstrated a stronger spatial relationship to C than was detectable in the full model. The individual species were then modeled again using basal area (m^2^/ha instead of Mg C/ha). Basal area was directly measured for each sample plot, and thus avoided the potential biases of allometric equations, which are typically developed from a small number of trees sampled from a single location rather than from across the species' range (as a result, such equations may be ill-suited for predicting volumetric relationships for individuals from other parts of the range).

Soil C (Mg C/ha to 1 m depth) was modeled in the same way as aboveground woody C using multiple linear regression, but with a different set of variable groups, including: 1) slope, solar radiation and profile curvature (a measure of the concavity or convexity of the 10 × 10 m cell); 2) land form (one of three topographic classifications: upland; valley/lowland; or terrace) and soil taxonomic group (the soil great group, from SSURGO); 3) ranch and habitat type; and 4) soil organic matter (from SSURGO). Different permutations of these variable groups were used to model soil C, and the best model was similarly selected using the AIC score. This model was then used to predict soil C across the landscape.

Ordination analysis was conducted using non-metric multi-dimensional scaling (NMDS) to examine the species and sample plot relationships to C stocks and environmental variables in multidimensional space. The variables included in the ordination were wood C (Mg C/ha), measured soil C (Mg C/ha to 1 m depth), slope, elevation, solar radiation, Normalized Difference Vegetation (or Water) Index (NDVI or NDWI, Summer or Winter), National Agriculture Imagery Program (NAIP) vegetation texture and soil organic matter, sand, clay and pH from the SURRGO database (Additional File 1: Appendix 4). All modeling and ordination analyses were conducted using R (R Development Core Team, 2009).

## Abbreviations

AIC: Akaike Information Criterion; C: Carbon; CAR: Climate Action Registry; DBH: Diameter at Breast Height; DCA: Detrended Correspondence Analysis; GHG: Greenhouse Gas; GIS: Geographic Information System; LiDAR: Light Detection and Ranging; NAIP: National Agriculture Imagery Program; NDVI: Normalized Difference Vegetation Index; NDWI: Normalized Difference Water Index; NMDS: Non-metric Multi-Dimensional Scaling; PAM: Partitioning around Medoids; SOC: Soil Organic Carbon; SSURGO: Soil Survey Geographic Database; USDA: United States Department of Agriculture.

## Competing interests

The authors declare that they have no competing interests.

## Authors' contributions

JNW collected the field data, conducted data analysis and was the lead writer; ADH and conducted most of the data analysis, ATO'G conducted the soil analysis; LAT, and KS helped design the research; RH collected the field data and contributed to the manuscript; GMG helped design the research and provided technical advice. LEJ was involved in all aspects of the manuscript including edits and revisions. All authors read and approved the final manuscript.

## Supplementary Material

Additional file 1 **Appendices to the manuscript**. Five appendices to the manuscript "Assessment of carbon in woody plants and soil across a vineyard-woodland landscape" are included in this file. The appendices are as follows: 1. Survey instrument and results of survey given to vineyard managers to determine vineyard management history. 2. Woody biomass data from sample plots in wildlands. 3. Woody species and genera encountered in wildland plots for which allometric equations to calculate aboveground woody biomass were available, listed with source of allometric equation used. 4. Ordination data and results. Includes sample sites and environmental variables used in modeling and ordination analysis, as well as a plot of the ordination results. 5. Vine carbon calculation. Includes regression data, graph of regression curve, and application of the resultant equation to estimate vine biomass as a function of age across the five ranches where the study was conducted. Click here for file
