# Supplementary material for: Assessment of carbon in woody plants and soil across a vineyard-woodland landscape
Source: Carbon Balance Manag. 2011 Nov 9;6:11. doi: 10.1186/1750-0680-6-11 (PMC3287142; doi:10.1186/1750-0680-6-11)
Supplement: Additional file 1 — Appendices to the manuscript. Five appendices to the manuscript "Assessment of carbon in woody plants and soil across a vineyard-woodland landscape" are included in this file. The appendices are as follows: 1. Survey instrument and results of survey given to vineyard managers to determine vineyard management history. 2. Woody biomass data from sample plots in wildlands. 3. Woody species and genera encountered in wildland plots for which allometric equations to calculate aboveground woody biomass were available, listed with source of allometric equation used. 4. Ordination data and results. Includes sample sites and environmental variables used in modeling and ordination analysis, as well as a plot of the ordination results. 5. Vine carbon calculation. Includes regression data, graph of regression curve, and application of the resultant equation to estimate vine biomass as a function of age across the five ranches where the study was conducted. [file 1750-0680-6-11-S1.DOC]

Appendices

Appendix 1. Survey instrument given to vineyard managers to determine management history.

1. Was the vineyard block planted in pastureland or perennial non-grape crops before Bonterra acquired it (yes=1; no=0)?
2. Was there annual mulching part of the management of this unit (yes=1; no=0)?
3. Is this block tilled every year (0) or every other year (1)?
4. Is compost applied to this block (yes=1; no=0)? If no, skip to question 6.
5. Is compost applied every year (1) or less frequently (0)?
6. Are cover crops planted on this block every year (1) or every other year (0)?


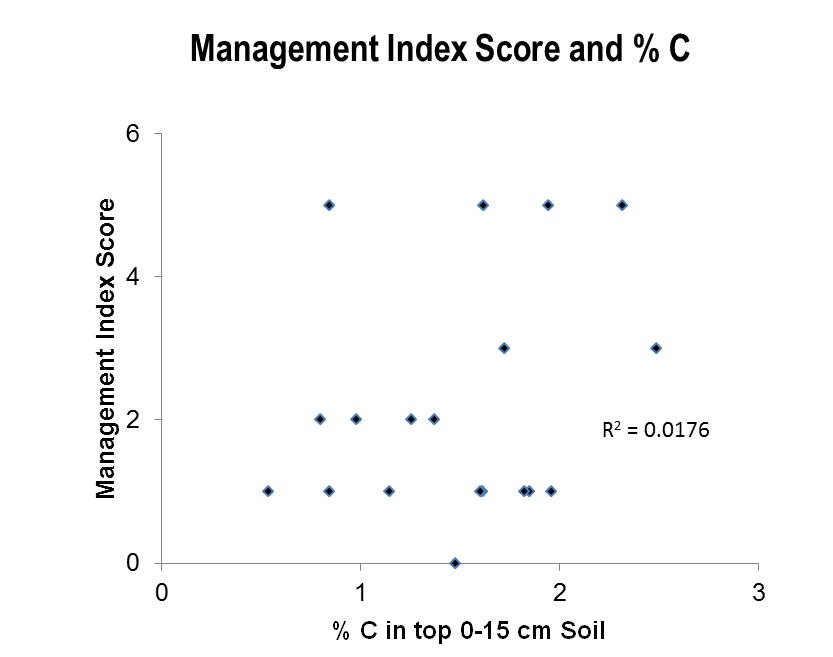


Appendix 2. Woody biomass data from sample plots in wildlands.

| **Ranch** | **Sample** | **# Species** | **# Individuals** | **Estimated Biomass (kg)** |
| --- | --- | --- | --- | --- |
| **Butler** | 1 | 3 | 48 | 1,435 |
|  | 2 | 5 | 15 | 2,971 |
|  | 3 | 4 | 30 | 3,892 |
|  | 4 | 3 | 10 | 4,769 |
|  | 5 | 3 | 21 | 4,907 |
|  | 6 | 3 | 20 | 5,084 |
|  | 7 | 2 | 25 | 64 |
|  | 8 | 3 | 23 | 6,090 |
|  | 9 | 3 | 26 | 3,695 |
|  | 10 | 3 | 13 | 4,913 |
|  | 11 | 4 | 29 | 7,681 |
|  | 12 | 5 | 19 | 1,546 |
|  | 13 | 3 | 41 | 3,005 |
|  | 14 | 3 | 57 | 2,394 |
|  | 15 | 4 | 19 | 4,637 |
|  | 16 | 5 | 19 | 5,468 |
|  | 17 | 3 | 16 | 2,530 |
|  | 18 | 3 | 16 | 4,434 |
|  | 19 | 3 | 24 | 2,381 |
|  | 20 | 5 | 25 | 2,394 |
|  | 21 | 5 | 28 | 5,033 |
|  | 22 | 5 | 38 | 1,844 |
|  | 23 | 3 | 23 | 4,878 |
| **Butler Average** |  | **3.6** | **25.4** | **3,741** |
| **Chalfont** | 1 | 2 | 20 | 3,215 |
|  | 2 | 2 | 21 | 3,975 |
|  | 3 | 4 | 30 | 1,906 |
|  | 4 | 1 | 23 | 2,917 |
| **Chalfont Average** |  | **2.3** | **23.5** | **3,003** |
| **Hooper** | 1 | 2 | 6 | 1,655 |
|  | 2 | 3 | 23 | 2,975 |
|  | 3 | 2 | 12 | 4,437 |
|  | 4 | 3 | 25 | 2,547 |
|  | 5 | 3 | 23 | 2,373 |
|  | 6 | 4 | 21 | 2,402 |
|  | 7 | 3 | 17 | 5,980 |
|  | 8 | 4 | 17 | 9,072 |
|  | 9 | 3 | 21 | 4,402 |
|  | 10 | 4 | 21 | 5,490 |
|  | 11 | 5 | 14 | 2,445 |
|  | 12 | 2 | 14 | 5,127 |
|  | 13 | 3 | 11 | 4,594 |
|  | 14 | 3 | 29 | 1,604 |
|  | 15 | 5 | 28 | 1,296 |
|  | 16 | 4 | 31 | 3,152 |
|  | 17 | 3 | 17 | 3,807 |
|  | 18 | 3 | 16 | 2,122 |
|  | 19 | 5 | 17 | 3,884 |
|  | 20 | 2 | 13 | 944 |
| **Hooper Average** |  | **3.3** | **18.8** | **3,515** |
| **McNab** | 1 | 2 | 6 | 2,173 |
|  | 2 | 1 | 2 | 1,679 |
|  | 3 | 2 | 6 | 1,119 |
|  | 4 | 3 | 4 | 601 |
|  | 5 | 2 | 8 | 2,854 |
|  | 6 | 2 | 3 | 4,361 |
|  | 7 | 1 | 3 | 875 |
|  | 8 | 1 | 11 | 1,859 |
|  | 9 | 2 | 22 | 1,831 |
|  | 10 | 3 | 4 | 2,486 |
|  | 11 | 2 | 8 | 3,680 |
|  | 12 | 3 | 9 | 6,510 |
|  | 13 | 1 | 4 | 1,088 |
|  | 14 | 3 | 15 | 3,125 |
|  | 15 | 2 | 6 | 6,683 |
|  | 16 | 3 | 7 | 5,735 |
|  | 17 | 2 | 14 | 5,088 |
|  | 18 | 4 | 26 | 3,765 |
|  | 19 | 5 | 18 | 2,802 |
|  | 20 | 3 | 24 | 2,742 |
|  | 21 | 3 | 18 | 3,028 |
|  | 22 | 1 | 3 | 1,972 |
|  | 23 | 1 | 3 | 4,602 |
|  | 24 | 2 | 62 | 1,318 |
|  | 25 | 2 | 19 | 2,968 |
|  | 26 | 3 | 16 | 2,823 |
|  | 27 | 2 | 24 | 15,651 |
|  | 28 | 5 | 6 | 2,126 |
|  | 29 | 4 | 21 | 2,580 |
| **McNab Total** |  | **2.4** | **12.8** | **3,384** |
| **Sundial** | 1 | 4 | 17 | 2,657 |
|  | 2 | 2 | 19 | 976 |
|  | 3 | 3 | 29 | 3,723 |
|  | 4 | 3 | 42 | 5,990 |
|  | 5 | 4 | 16 | 2,138 |
|  | 6 | 2 | 3 | 4,905 |
|  | 7 | 1 | 12 | 10,557 |
|  | 8 | 2 | 5 | 3,037 |
|  | 9 | 3 | 20 | 5,876 |
|  | 10 | 2 | 8 | 1,877 |
|  | 11 | 2 | 15 | 2,495 |
|  | 12 | 1 | 1 | 6,553 |
|  | 13 | 2 | 5 | 6,849 |
|  | 14 | 2 | 19 | 4,359 |
|  | 15 | 4 | 28 | 7,171 |
|  | 16 | 3 | 16 | 11,398 |
|  | 17 | 3 | 13 | 1,731 |
| **Sundial Average** |  | **2.5** | **15.8** | **4,841** |

Appendix 3. Woody species and genera encountered in wildland plots for which allometric equations to calculate aboveground woody biomass were available, listed with source of allometric equation used. Equation numbers are listed in parentheses where appropriate.

| **Common Name** | **Scientific Name** | **Family** | **Equation Source** |
| --- | --- | --- | --- |
| Bigleaf Maple | *Acer macrophyllum* | Aceraceae | Jenkins et al. 1984 (1000) |
| Box Elder | *Acer negundo* | Aceraceae | Jenkins et al. 1984 (970) |
| Chamise | *Adenostoma fasciculatum* | Rosaceae | McGinnis & Keeley 2009 |
| California Buckeye | *Aesculus californica* | Hippocastanaceae | Jenkins et al. 1984 (541) |
| Alder sp. | *Alnus sp.* | Betulaceae | Jenkins et al. 1984 (316) |
| Pacific Madrone | *Arbutus menziesii* | Ericaceae | Pillsbury & Kirkley 1984 |
| N. California manzanita | *Arctostaphylos manzanita* | Ericaceae | McGinnis & Keeley 2009 |
| Whiteleaf manzanita | *Arctostaphylos viscida* | Ericaceae | McGinnis & Keeley 2009 |
| Manzanita sp. | *Arctostaphylos sp.* | Ericaceae | McGinnis & Keeley 2009 |
| Ceonothus sp. | *Ceonothus sp.* | Rhamnaceae | McGinnis & Keeley 2009 |
| Mountain Mahogany | *Cercocarpus betuloides* | Rosaceae | Jenkins et al. 1984 (475) |
| Golden Chinquapin | *Chrysolepis chrysophylla* | Fagaceae | Pillsbury & Kirkley 1984 |
| Oregon Ash | *Fraxinus latifolia* | Oleaceae | Jenkins et al. 1984 (541) |
| Toyon | *Heteromeles arbutifolia* | Rosaceae | McGinnis & Keeley 2009 |
| California Walnut | *Juglans californica* | Juglandaceae | Jenkins et al. 1984 (951) |
| Fremont Cottonwood | *Populus fremontii* | Salicaceae | Jenkins et al. 1984 (740) |
| Cottonwood | *Populus sp.* | Salicaceae | Jenkins et al. 1984 (740) |
| Douglas Fir | *Pseudotsuga menziesii* | Pinaceae | Jenkins et al. 1984 (202) |
| Coast Live Oak | *Quercus agrifolia* | Fagaceae | Pillsbury & Kirkley 1984 |
| Canyon Live Oak | *Quercus chrysolepis* | Fagaceae | Pillsbury & Kirkley 1984 |
| Blue Oak | *Quercus douglasii* | Fagaceae | Pillsbury & Kirkley 1984 |
| Oregon White Oak | *Quercus garryana* | Fagaceae | Pillsbury & Kirkley 1984 |
| Black Oak | *Quercus kelloggii* | Fagaceae | Pillsbury & Kirkley 1984 |
| Valley Oak | *Quercus lobata* | Fagaceae | Pillsbury & Kirkley 1984 |
| Interior Live Oak | *Quercus wislizenii* | Fagaceae | Pillsbury & Kirkley 1984 |
| Oak | *Quercus sp.* | Fagaceae | Pillsbury & Kirkley 1984 |
| Willow | *Salix sp.* | Salicaceae | Jenkins et al. 1984 (316) |
| California Bay Laurel | *Umbellularia californica* | Lauraceae | Pillsbury & Kirkley 1984 |
| Unknown Hardwoods |  |  | Jenkins et al. 1984 (1000) |

Appendix 4. Ordination data and results

1. Sample sites and environmental variables used in modeling and ordination analysis. Columns include elevation (meters); irradiation (j/m^2^/day); landcover (MHW=Mixed Hardwood; VRI=Vineyard Riparian; MCH=Mixed Conifer-Hardwood; Urb=Urban (near buildings)); slope (%); Normalized Difference Vegetation (or Water) Index (NDVI or NDWI, Summer or Winter); soil sand, clay, organic matter (%), and pH; National Agriculture Imagery Program (NAIP) texture, ranch, soil great group, and measured carbon (Mg C/ha).

|  | Elev | Irrad. | Landcvr | Slope | S_NDVI | S_NDWI | W_NDVI | W_NDWI | Sand | Clay | OM | pH | NAIP | Ranch | GreatGroup | C |
| --- | --- | --- | --- | --- | --- | --- | --- | --- | --- | --- | --- | --- | --- | --- | --- | --- |
| 1 | 252 | 2,099,381 | MHW | 32.3 | 0.241 | 0.264 | 0.381 | 0.094 | 37.5 | 24.7 | 1.45 | 6.25 | 132.8 | Bonterra | Haploxeralfs | 36.2 |
| 2 | 293 | 2,441,136 | MHW | 40.9 | 0.325 | 0.309 | 0.356 | 0.119 | 37.5 | 24.7 | 1.45 | 6.25 | 214.9 | Bonterra | Haploxeralfs | 30.3 |
| 3 | 295 | 1,868,390 | MHW | 36.2 | 0.343 | 0.318 | 0.327 | 0.127 | 37.5 | 24.7 | 1.45 | 6.25 | 196.5 | Bonterra | Haploxeralfs | 32.6 |
| 4 | 226 | 2,021,615 | MHW | 24.6 | 0.361 | 0.380 | 0.349 | 0.067 | 37.5 | 24.7 | 1.45 | 6.25 | 209.9 | Bonterra | Haploxeralfs | 22.7 |
| 5 | 239 | 2,597,864 | MHW | 31.8 | 0.506 | 0.412 | 0.347 | 0.063 | 37.5 | 24.7 | 1.45 | 6.25 | 158.4 | Bonterra | Haploxeralfs | 48.9 |
| 6 | 233 | 2,560,320 | MHW | 29.5 | 0.506 | 0.412 | 0.347 | 0.063 | 37.5 | 24.7 | 1.45 | 6.25 | 214.9 | Bonterra | Haploxeralfs | 74.0 |
| 7 | 207 | 2,519,408 | MHW | 25.5 | 0.494 | 0.457 | 0.393 | 0.109 | 37.5 | 24.7 | 1.45 | 6.25 | 181.4 | Bonterra | Haploxeralfs | 14.6 |
| 8 | 185 | 2,157,708 | VRI | 0.5 | 0.218 | 0.200 | 0.706 | 0.500 | 26.2 | 33.3 | 1.41 | 7.20 | 187.7 | Bonterra | Argixerolls | 34.5 |
| 9 | 186 | 2,147,268 | VRI | 1.1 | 0.510 | 0.408 | 0.607 | 0.295 | 26.2 | 33.3 | 1.41 | 7.20 | 269.4 | Bonterra | Argixerolls | 51.3 |
| 10 | 400 | 1,758,583 | NA | 27.6 | 0.617 | 0.502 | 0.627 | 0.352 | 37.5 | 24.7 | 1.45 | 6.25 | 265.3 | NA | Haploxeralfs | 44.0 |
| 11 | 351 | 1,466,746 | NA | 31.9 | 0.646 | 0.537 | 0.458 | 0.186 | 37.5 | 24.7 | 1.45 | 6.25 | 154.0 | NA | Haploxeralfs | 61.6 |
| 12 | 326 | 1,771,698 | NA | 24.0 | 0.706 | 0.544 | 0.458 | 0.111 | 37.5 | 24.7 | 1.45 | 6.25 | 129.5 | NA | Haploxeralfs | 109.9 |
| 13 | 310 | 1,799,806 | NA | 24.0 | 0.726 | 0.584 | 0.368 | 0.072 | 37.5 | 24.7 | 1.45 | 6.25 | 139.2 | NA | Haploxeralfs | 30.0 |
| 14 | 332 | 1,269,493 | NA | 33.4 | 0.656 | 0.552 | 0.182 | 0.083 | 37.5 | 24.7 | 1.45 | 6.25 | 126.6 | NA | Haploxeralfs | 52.9 |
| 15 | 358 | 1,513,715 | NA | 24.2 | 0.692 | 0.542 | 0.800 | 0.636 | 37.5 | 24.7 | 1.45 | 6.25 | 238.3 | NA | Haploxeralfs | 114.8 |
| 16 | 297 | 1,916,241 | NA | 13.7 | 0.742 | 0.602 | 0.413 | 0.182 | 41.6 | 27.0 | 1.58 | 6.25 | 204.0 | NA | Argixerolls | 110.9 |
| 17 | 287 | 1,813,195 | NA | 14.6 | 0.698 | 0.586 | 0.458 | 0.365 | 41.6 | 27.0 | 1.58 | 6.25 | 114.5 | NA | Argixerolls | 89.8 |
| 18 | 268 | 1,660,359 | NA | 18.0 | 0.636 | 0.500 | 0.436 | 0.258 | 41.6 | 27.0 | 1.58 | 6.25 | 216.5 | NA | Argixerolls | 64.6 |
| 19 | 244 | 1,597,616 | MHW | 20.3 | 0.635 | 0.571 | 0.725 | 0.504 | 41.6 | 27.0 | 1.58 | 6.25 | 144.3 | Bonterra | Argixerolls | 52.2 |
| 20 | 232 | 1,771,552 | MHW | 14.4 | 0.533 | 0.438 | 0.487 | 0.254 | 41.6 | 27.0 | 1.58 | 6.25 | 196.7 | Bonterra | Argixerolls | 50.9 |
| 21 | 237 | 1,756,111 | MHW | 16.1 | 0.556 | 0.434 | 0.429 | 0.184 | 41.6 | 27.0 | 1.58 | 6.25 | 202.1 | Bonterra | Argixerolls | 50.7 |
| 22 | 227 | 1,441,256 | NA | 25.2 | 0.552 | 0.399 | 0.273 | 0.191 | 41.6 | 27.0 | 1.58 | 6.25 | 290.3 | NA | Argixerolls | 32.9 |
| 23 | 223 | 2,029,415 | MHW | 7.1 | 0.400 | 0.350 | 0.194 | 0.132 | 41.6 | 27.0 | 1.58 | 6.25 | 217.9 | Bonterra | Argixerolls | 76.7 |
| 24 | 195 | 2,153,653 | VRI | 0.5 | 0.361 | 0.407 | 0.169 | 0.169 | 26.2 | 33.3 | 1.41 | 7.20 | 200.2 | Bonterra | Argixerolls | 23.8 |
| 25 | 192 | 2,016,498 | VRI | 7.6 | 0.373 | 0.401 | 0.402 | 0.224 | 26.2 | 33.3 | 1.41 | 7.20 | 153.1 | Bonterra | Argixerolls | 62.4 |
| 26 | 189 | 2,140,477 | VRI | 0.8 | 0.569 | 0.486 | 0.432 | 0.156 | 26.2 | 33.3 | 1.41 | 7.20 | 254.9 | Bonterra | Argixerolls | 50.8 |
| 27 | 269 | 1,797,358 | MHW | 16.5 | 0.637 | 0.498 | 0.250 | 0.111 | 41.6 | 27.0 | 1.58 | 6.25 | 191.5 | Bonterra | Argixerolls | 189.1 |
| 28 | 312 | 1,651,761 | MHW | 24.2 | 0.602 | 0.498 | 0.552 | 0.385 | 41.6 | 27.0 | 1.58 | 6.25 | 175.6 | Bonterra | Argixerolls | 43.9 |
| 29 | 244 | 1,822,177 | MHW | 12.2 | 0.633 | 0.561 | 0.717 | 0.444 | 41.6 | 27.0 | 1.58 | 6.25 | 166.5 | Bonterra | Argixerolls | 74.7 |
| 30 | 262 | 1,962,751 | MHW | 9.1 | 0.457 | 0.366 | 0.421 | 0.161 | 41.6 | 27.0 | 1.58 | 6.25 | 246.1 | Bonterra | Argixerolls | 20.0 |
| 31 | 487 | 1,835,807 | MHW | 18.7 | 0.648 | 0.510 | 0.405 | 0.333 | 37.5 | 26.9 | 1.11 | 6.29 | 156.5 | Butler | Argixerolls | 24.1 |
| 32 | 490 | 2,189,669 | MHW | 6.7 | 0.600 | 0.513 | 0.461 | 0.167 | 37.5 | 26.9 | 1.11 | 6.29 | 162.1 | Butler | Argixerolls | 52.3 |
| 33 | 503 | 2,358,404 | MHW | 24.2 | 0.640 | 0.584 | 0.821 | 0.560 | 37.5 | 26.9 | 1.11 | 6.29 | 152.1 | Butler | Argixerolls | 67.6 |
| 34 | 507 | 2,061,343 | MHW | 12.1 | 0.552 | 0.468 | 0.495 | 0.224 | 37.5 | 26.9 | 1.11 | 6.29 | 165.2 | Butler | Argixerolls | 84.7 |
| 35 | 524 | 1,762,953 | MHC | 16.5 | 0.424 | 0.353 | 0.525 | 0.184 | 30.9 | 42.6 | 1.38 | 5.48 | 130.4 | Butler | Palexeralfs | 82.0 |
| 36 | 652 | 1,574,258 | MHW | 22.3 | 0.733 | 0.602 | 0.947 | 0.480 | 37.5 | 24.7 | 1.60 | 6.25 | 191.7 | Butler | Haploxeralfs | 89.0 |
| 37 | 652 | 2,325,204 | MCH | 5.1 | 0.438 | 0.406 | 0.500 | 0.346 | 53.3 | 16.8 | 0.91 | 6.10 | 161.6 | Butler | Xerochrepts | 1.1 |
| 38 | 625 | 1,982,721 | MHW | 16.4 | 0.717 | 0.619 | 0.741 | 0.528 | 37.5 | 24.7 | 1.60 | 6.25 | 197.7 | Butler | Haploxeralfs | 101.5 |
| 39 | 579 | 1,684,505 | MHW | 20.1 | 0.707 | 0.606 | 0.577 | 0.390 | 37.5 | 24.7 | 1.60 | 6.25 | 140.2 | Butler | Haploxeralfs | 67.0 |
| 40 | 591 | 1,375,360 | MHW | 28.3 | 0.706 | 0.586 | 0.436 | 0.302 | 37.5 | 24.7 | 1.60 | 6.25 | 179.4 | Butler | Haploxeralfs | 86.3 |
| 41 | 601 | 2,000,953 | MHC | 10.2 | 0.554 | 0.474 | 0.519 | 0.302 | 38.4 | 27.0 | 1.69 | 6.31 | 210.3 | Butler | Haploxeralfs | 128.0 |
| 42 | 661 | 1,504,323 | MHC | 24.3 | 0.663 | 0.606 | 0.793 | 0.625 | 38.4 | 27.0 | 1.69 | 6.31 | 231.8 | Butler | Haploxeralfs | 26.7 |
| 43 | 636 | 1,879,825 | MHC | 20.8 | 0.583 | 0.494 | 0.750 | 0.482 | 38.4 | 27.0 | 1.69 | 6.31 | 176.9 | Butler | Haploxeralfs | 53.7 |
| 44 | 478 | 1,981,371 | MHC | 47.2 | 0.539 | 0.521 | 0.750 | 0.458 | 38.4 | 27.0 | 1.69 | 6.31 | 281.0 | Butler | Haploxeralfs | 40.4 |
| 45 | 544 | 1,905,180 | MHW | 23.8 | 0.561 | 0.424 | 0.581 | 0.322 | 37.5 | 24.7 | 1.60 | 6.25 | 161.3 | Butler | Haploxeralfs | 83.8 |
| 46 | 538 | 1,837,388 | MHW | 20.1 | 0.719 | 0.590 | 0.604 | 0.304 | 37.5 | 24.7 | 1.60 | 6.25 | 173.8 | Butler | Haploxeralfs | 109.8 |
| 47 | 658 | 2,508,890 | MHW | 15.6 | 0.527 | 0.493 | 0.536 | 0.297 | 37.5 | 24.7 | 1.60 | 6.25 | 167.9 | Butler | Haploxeralfs | 45.5 |
| 48 | 657 | 2,585,115 | MHW | 21.9 | 0.574 | 0.486 | 0.544 | 0.257 | 37.5 | 24.7 | 1.60 | 6.25 | 218.8 | Butler | Haploxeralfs | 77.7 |
| 49 | 369 | 1,257,225 | MHC | 43.6 | 0.722 | 0.687 | 0.698 | 0.659 | 38.4 | 27.0 | 1.69 | 6.31 | 165.0 | Butler | Haploxeralfs | 49.2 |
| 50 | 495 | 1,653,580 | MHW | 30.5 | 0.442 | 0.405 | 0.308 | 0.308 | 37.5 | 26.9 | 1.11 | 6.29 | 109.9 | Butler | Argixerolls | 40.2 |
| 51 | 451 | 2,423,954 | MHW | 26.9 | 0.261 | 0.285 | 0.441 | 0.212 | 53.3 | 16.8 | 0.91 | 6.10 | 146.8 | Butler | Xerochrepts | 88.7 |
| 52 | 458 | 1,584,864 | MHW | 22.5 | 0.665 | 0.544 | 0.650 | 0.467 | 53.3 | 16.8 | 0.91 | 6.10 | 208.6 | Butler | Xerochrepts | 31.3 |
| 53 | 610 | 1,525,374 | MHW | 23.1 | 0.624 | 0.504 | 0.371 | 0.103 | 37.5 | 24.7 | 1.60 | 6.25 | 171.2 | Butler | Haploxeralfs | 85.5 |
| 54 | 168 | 2,179,888 | VRI | 0.3 | 0.723 | 0.595 | 0.380 | 0.150 | 70.8 | 8.1 | 2.50 | 7.00 | 213.2 | Chalfont | Xerofluvents | 63.2 |
| 55 | 168 | 2,184,123 | VRI | 0.3 | 0.692 | 0.620 | 0.354 | 0.171 | 70.8 | 8.1 | 2.50 | 7.00 | 212.5 | Chalfont | Xerofluvents | 76.8 |
| 56 | 168 | 2,184,567 | VRI | 0.5 | 0.686 | 0.600 | 0.420 | 0.164 | 70.8 | 8.1 | 2.50 | 7.00 | 211.7 | Chalfont | Xerofluvents | 39.3 |
| 57 | 168 | 2,183,137 | VRI | 0.4 | 0.659 | 0.593 | 0.442 | 0.230 | 70.8 | 8.1 | 2.50 | 7.00 | 148.3 | Chalfont | Xerofluvents | 52.3 |
| 58 | 286 | 2,129,985 | MHW | 4.7 | 0.325 | 0.286 | 0.443 | 0.120 | 44.5 | 23.0 | 1.61 | 6.18 | 211.2 | Hooper | Xerochrepts | 27.6 |
| 59 | 308 | 2,059,010 | MHW | 11.3 | 0.594 | 0.526 | 0.810 | 0.522 | 44.5 | 23.0 | 1.61 | 6.18 | 251.2 | Hooper | Xerochrepts | 53.0 |
| 60 | 276 | 2,046,975 | MHW | 11.7 | 0.511 | 0.471 | 0.600 | 0.436 | 44.5 | 23.0 | 1.61 | 6.18 | 162.5 | Hooper | Xerochrepts | 74.0 |
| 61 | 279 | 1,956,613 | MHW | 7.0 | 0.430 | 0.355 | 0.543 | 0.367 | 41.6 | 27.0 | 1.58 | 6.25 | 169.1 | Hooper | Argixerolls | 45.2 |
| 62 | 294 | 1,577,962 | MHW | 20.5 | 0.652 | 0.598 | 0.705 | 0.465 | 41.6 | 27.0 | 1.58 | 6.25 | 134.0 | Hooper | Argixerolls | 39.6 |
| 63 | 315 | 1,813,415 | MHW | 14.6 | 0.536 | 0.440 | 0.509 | 0.211 | 41.6 | 27.0 | 1.58 | 6.25 | 176.9 | Hooper | Argixerolls | 43.5 |
| 64 | 289 | 2,184,291 | MHW | 19.2 | 0.534 | 0.484 | 0.678 | 0.377 | 41.6 | 27.0 | 1.58 | 6.25 | 173.0 | Hooper | Argixerolls | 101.8 |
| 65 | 234 | 2,391,348 | MHW | 14.4 | 0.664 | 0.589 | 0.632 | 0.406 | 40.5 | 28.5 | 1.03 | 6.74 | 148.5 | Hooper | Argixerolls | 170.9 |
| 66 | 262 | 1,593,149 | MHW | 24.3 | 0.481 | 0.449 | 0.563 | 0.538 | 44.5 | 23.0 | 1.61 | 6.18 | 108.8 | Hooper | Xerochrepts | 75.8 |
| 67 | 288 | 1,811,165 | MHW | 20.8 | 0.646 | 0.570 | 0.662 | 0.521 | 44.5 | 23.0 | 1.61 | 6.18 | 152.5 | Hooper | Xerochrepts | 91.5 |
| 68 | 281 | 1,417,685 | NA | 28.0 | 0.549 | 0.447 | 0.560 | 0.322 | 44.5 | 23.0 | 1.61 | 6.18 | 104.3 | NA | Xerochrepts | 41.6 |
| 69 | 250 | 2,300,611 | MHW | 12.3 | 0.502 | 0.414 | 0.554 | 0.297 | 40.5 | 28.5 | 1.03 | 6.74 | 189.2 | Hooper | Argixerolls | 86.1 |
| 70 | 260 | 2,224,265 | MHW | 2.1 | 0.568 | 0.505 | 0.577 | 0.390 | 65.7 | 10.9 | 0.96 | 6.89 | 189.7 | Hooper | Haploxerolls | 77.1 |
| 71 | 273 | 2,202,056 | MHW | 5.5 | 0.489 | 0.398 | 0.262 | 0.012 | 44.5 | 23.0 | 1.61 | 6.18 | 203.5 | Hooper | Xerochrepts | 41.8 |
| 72 | 293 | 2,055,827 | MHW | 13.2 | 0.598 | 0.532 | 0.612 | 0.403 | 44.5 | 23.0 | 1.61 | 6.18 | 210.5 | Hooper | Xerochrepts | 21.9 |
| 73 | 303 | 1,625,148 | N/A | 23.7 | 0.494 | 0.485 | 0.609 | 0.370 | 44.5 | 23.0 | 1.61 | 6.18 | 137.8 | Hooper | Xerochrepts | 53.8 |
| 74 | 309 | 1,587,399 | MHW | 32.3 | 0.578 | 0.532 | 0.793 | 0.529 | 41.6 | 27.0 | 1.58 | 6.25 | 218.6 | Hooper | Argixerolls | 63.5 |
| 75 | 405 | 1,583,960 | MHW | 22.9 | 0.648 | 0.515 | 0.410 | 0.162 | 41.6 | 27.0 | 1.58 | 6.25 | 130.2 | Hooper | Argixerolls | 41.1 |
| 76 | 279 | 1,762,608 | MHW | 16.8 | 0.622 | 0.607 | 0.743 | 0.564 | 41.6 | 27.0 | 1.58 | 6.25 | 123.5 | Hooper | Argixerolls | 68.3 |
| 77 | 289 | 2,464,880 | MHW | 34.0 | 0.448 | 0.440 | 0.615 | 0.400 | 44.5 | 23.0 | 1.61 | 6.18 | 114.9 | Hooper | Xerochrepts | 18.7 |
| 78 | 155 | 2,191,688 | VRI | 1.0 | 0.633 | 0.541 | 0.526 | 0.196 | 26.2 | 33.3 | 1.41 | 7.20 | 189.7 | Sundial | Argixerolls | 52.9 |
| 79 | 153 | 2,189,778 | VRI | 0.4 | 0.633 | 0.572 | 0.321 | 0.061 | 0.0 | 0.0 | 0.00 | 0.00 | 198.3 | Sundial | NA | 31.8 |
| 80 | 153 | 2,177,309 | VRI | 0.0 | 0.388 | 0.427 | 0.500 | 0.145 | 70.8 | 8.1 | 2.50 | 7.00 | 160.4 | Sundial | Xerofluvents | 66.5 |
| 81 | 153 | 2,177,093 | VRI | 0.0 | 0.287 | 0.363 | 0.449 | 0.296 | 44.3 | 15.0 | 2.50 | 7.00 | 188.8 | Sundial | Haploxerolls | 121.4 |
| 82 | 153 | 2,184,078 | VRI | 0.3 | 0.373 | 0.367 | 0.326 | 0.208 | 44.3 | 15.0 | 2.50 | 7.00 | 207.9 | Sundial | Haploxerolls | 40.9 |
| 83 | 208 | 2,327,781 | MHW | 14.8 | 0.523 | 0.498 | 0.729 | 0.523 | 30.9 | 42.6 | 1.38 | 5.48 | 190.1 | Sundial | Palexeralfs | 83.4 |
| 84 | 199 | 2,304,534 | MHW | 7.3 | 0.278 | 0.251 | 0.440 | 0.091 | 30.9 | 42.6 | 1.38 | 5.48 | 216.0 | Sundial | Palexeralfs | 175.9 |
| 85 | 215 | 2,240,789 | MHW | 14.8 | 0.292 | 0.239 | 0.436 | 0.087 | 42.9 | 25.3 | 0.74 | 6.19 | 120.9 | Sundial | Haploxeralfs | 51.9 |
| 86 | 207 | 2,132,637 | MHW | 3.2 | 0.530 | 0.450 | 0.486 | 0.284 | 32.0 | 35.3 | 1.03 | 7.20 | 186.3 | Sundial | Argixerolls | 98.8 |
| 87 | 204 | 1,753,177 | MHW | 18.4 | 0.690 | 0.586 | 0.822 | 0.496 | 30.9 | 42.6 | 1.38 | 5.48 | 155.0 | Sundial | Palexeralfs | 31.6 |
| 88 | 197 | 1,637,664 | MHW | 21.2 | 0.625 | 0.546 | 0.646 | 0.529 | 30.9 | 42.6 | 1.38 | 5.48 | 178.4 | Sundial | Palexeralfs | 43.1 |
| 89 | 183 | 2,459,093 | MHW | 18.3 | 0.374 | 0.367 | 0.410 | 0.154 | 30.9 | 42.6 | 1.38 | 5.48 | 190.7 | Sundial | Palexeralfs | 114.5 |
| 90 | 161 | 2,188,154 | URB | 1.5 | 0.556 | 0.577 | 0.748 | 0.538 | 36.9 | 27.6 | 1.03 | 6.55 | 170.9 | Sundial | Argixerolls | 72.7 |
| 91 | 153 | 2,181,829 | VRI | 0.3 | 0.709 | 0.604 | 0.333 | 0.043 | 44.3 | 15.0 | 2.50 | 7.00 | 199.7 | Sundial | Haploxerolls | 147.8 |
| 92 | 184 | 2,340,320 | MHW | 6.5 | 0.319 | 0.279 | 0.397 | 0.135 | 40.5 | 28.5 | 1.03 | 6.74 | 192.3 | Sundial | Argixerolls | 190.0 |
| 93 | 184 | 2,113,373 | MHW | 18.7 | 0.418 | 0.452 | 0.518 | 0.273 | 40.5 | 28.5 | 1.03 | 6.74 | 158.2 | Sundial | Argixerolls | 31.2 |

1. Ordination of woody species by environmental variables (see Appendix 6) using non-metric multidimensional scaling (NMDS) along the primary (horizontal) and secondary (vertical) axes. Species are abbreviated according to the first two letters of the genus combined with the first two letters of the specific epithet, with ¨SP¨ used where species identity was uncertain. Environmental variables are elevation in meters (elev); irradiation in joules/meter^2^/day (irrad); slope in percent; normalized difference vegetation (or water) index (ndvi or ndwi, summer (S) or winter (W)); pH and percent sand, clay, and organic matter (om) in soil (from USDA SSURGO); National Agriculture Imagery Program texture (naiptext), and total stored carbon.


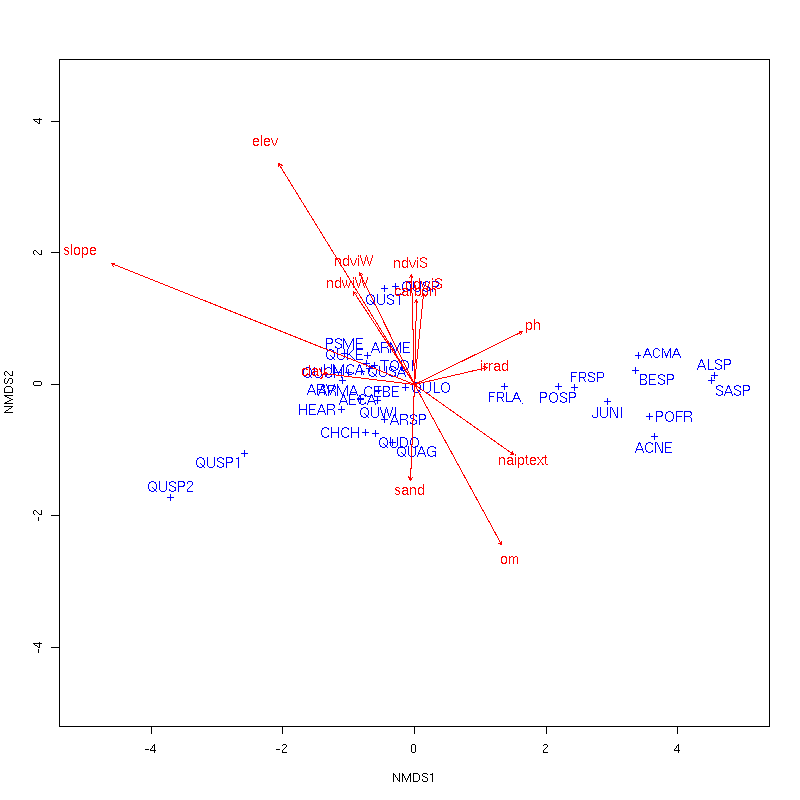


Appendix 5. Vine carbon calculation

A. Regression of vine volume on age, based on samples from different aged vineyard tracts

| Vineyard/Tract | # Individuals (n) | Age (yrs) | Avg Wood Vol (cm^3)* |
| --- | --- | --- | --- |
| Butler 1 | 6 | 7 | 1,202 |
| Butler 2 | 6 | 7 | 1,409 |
| Butler 3 | 5 | 7 | 2,005 |
| Butler 4 | 5 | 8 | 2,427 |
| Chalfont-Ledford 1 | 6 | 19 | 9,522 |
| Sundial 1 | 6 | 8 | 2,975 |
| Sundial 2 | 6 | 21 | 8,740 |
| Sundial 3 | 6 | 21 | 7,577 |
| Sundial 4 | 6 | 2 | 510 |
| Sundial 5 | 6 | 12 | 4,240 |
| Sundial 6 | 6 | 13 | 11,878 |
| Sundial 7 | 6 | 10 | 4,173 |
| Sundial 8 | 6 | 22 | 12,434 |
| Sundial 9 | 6 | 14 | 6,245 |
| Sundial 10 | 6 | 22 | 12,045 |
| Sundial 11 | 6 | 5 | 2,223 |
| Sundial 12 | 6 | 15 | 5,258 |
| Sundial 13 | 6 | 15 | 6,667 |
| Sundial 14 | 6 | 13 | 7,797 |
| Sundial 15 | 6 | 14 | 6,039 |
| McNab 1 | 6 | 11 | 5,087 |
| McNab 2 | 6 | 11 | 7,829 |
| McNab 3 | 6 | 13 | 5,059 |
| McNab 4 | 6 | 13 | 5,481 |
| McNab 5 | 6 | 13 | 6,361 |
| McNab 6 | 6 | 14 | 5,304 |
| McNab 7 | 6 | 15 | 5,170 |
| McNab 8 | 6 | 15 | 6,003 |
| McNab 9 | 6 | 15 | 5,840 |
| * Estimate based on trunk and two principal cordons only (see text). | | | |


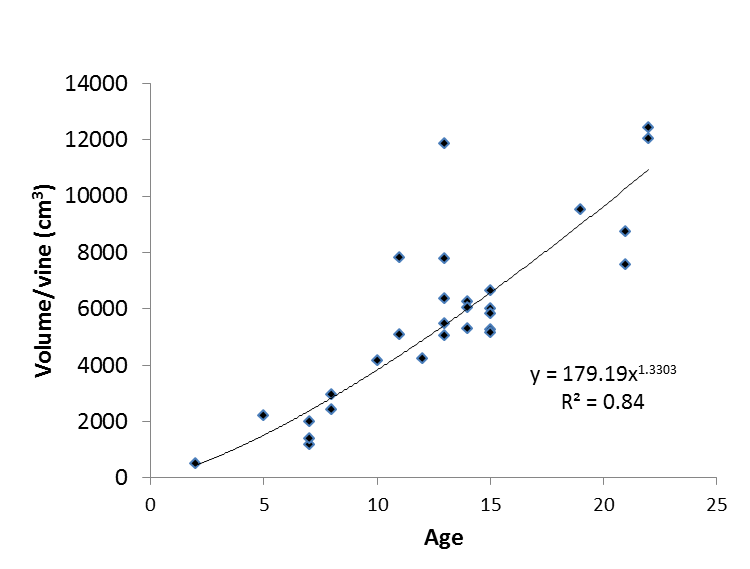


B. Regression of vine volume on vine age (years). Regression equation is an exponential function that has not been evaluated for vines older than 23 years of age.

C) Estimates for above-ground carbon in vineyard tracts.

| **Tract/Ranch** | **Vine Age** | **HA** | **Kg C/vine** | **C Mg/HA** | **C Mg/tract** |
| --- | --- | --- | --- | --- | --- |
| **1** | 8 | 21.1 | 2.3 | 2.3 | 51.3 |
| **2** | 17 | 8.1 | 3.1 | 4.2 | 33.8 |
| **3** | 18 | 10.8 | 3.3 | 4.5 | 48.6 |
| **4** | 19 | 10.6 | 3.6 | 4.8 | 51.4 |
| **Chalfont/Ledford** |  | **50.6** | **12.3** | **3.6** | **185.1** |
| **1** | 7 | 24.7 | 14.3 | 2.1 | 50.8 |
| **2** | 8 | 15.0 | 9.1 | 2.7 | 42.0 |
| **Butler** |  | **39.7** | **23.4** | **2.3** | **92.9** |
| **1** | 7 | 3.0 | 3.8 | 2.6 | 7.7 |
| **2** | 11 | 8.7 | 3.5 | 3.0 | 32.8 |
| **3** | 13 | 14.6 | 6.5 | 4.9 | 70.9 |
| **4** | 14 | 15.7 | 7.2 | 5.1 | 82.4 |
| **5** | 15 | 8.9 | 10.5 | 5.3 | 51.7 |
| **6** | 33 | 3.5 | 7.5 | 9.6 | 34.1 |
| **McNab** |  | **54.5** | **39.0** | **4.5** | **279.5** |
| **1** | 2 | 2.3 | 0.2 | 0.5 | 1.1 |
| **2** | 5 | 12.2 | 1.8 | 1.2 | 14.2 |
| **3** | 8 | 8.8 | 3.4 | 2.1 | 18.3 |
| **4** | 9 | 11.0 | 9.3 | 2.6 | 28.8 |
| **5** | 10 | 10.7 | 1.5 | 2.9 | 31.5 |
| **6** | 12 | 3.1 | 2.0 | 3.0 | 9.4 |
| **7** | 13 | 1.2 | 4.3 | 3.5 | 4.0 |
| **8** | 14 | 4.9 | 7.2 | 3.7 | 18.2 |
| **9** | 15 | 15.6 | 2.6 | 4.0 | 62.8 |
| **10** | 17 | 0.4 | 3.1 | 13.9 | 5.2 |
| **11** | 18 | 0.3 | 3.3 | 9.1 | 2.7 |
| **12** | 21 | 27.4 | 4.1 | 7.4 | 202.0 |
| **13** | 22 | 7.4 | 8.7 | 5.5 | 41.2 |
| **14** | 23 | 7.6 | 4.6 | 9.1 | 69.5 |
| **15** | 25 | 3.0 | 5.2 | 5.8 | 17.2 |
| **16** | 34 | 1.7 | 7.8 | 10.0 | 17.1 |
| **Sundial** |  | **117.6** | **69.3** | **4.1** | **543.1** |
| **1** | 1 | 38.2 | 0.4 | 0.2 | 7.4 |
| **2** | 3 | 17.9 | 0.9 | 0.8 | 14.9 |
| **3** | 5 | 15.7 | 0.6 | 1.2 | 19.3 |
| **4** | 6 | 15.9 | 0.8 | 2.5 | 40.0 |
| **Hooper** |  | **87.6** | **2.7** | **0.7** | **81.5** |
